# The perceived onset position of a moving target: Effects of trial contexts are evoked by different attentional allocations

**DOI:** 10.3758/s13414-012-0397-6

**Published:** 2012-11-14

**Authors:** Jochen Müsseler, Jens Tiggelbeck

**Affiliations:** 1 RWTH Aachen University, Aachen, Germany; 2 Work and Cognitive Psychology, RWTH Aachen University, Jägerstr. 17-19, 52066 Aachen, Germany

**Keywords:** Attention, Space perception, Localization, Position judgments, Onset position, Moving stimuli, Motion, Fröhlich effect

## Abstract

Previous studies have shown that the localization of the perceived onset position of a moving target varies with the trial context. When the moving target appeared at predictable positions to the left or right of fixation (constant context), localization judgments of the perceived onset positions were essentially displaced in motion direction (Fröhlich effect). In contrast, when the target appeared at unpredictable positions in the visual field (random context), localization judgments were at least drastically reduced. Four explanations of this influence of trial context on localization judgments were examined in three experiments. Findings ruled out an overcompensation mechanism effective in random-context conditions, a predictive mechanism effective in constant-context conditions and a detrimental mechanism originating from more trial repetitions in constant-context conditions. Instead, the results indicated that different attentional allocations are responsible for the localization differences. They also demonstrated that attentional mechanisms are at the basis of the Fröhlich effect.

## Introduction

Perception of the initial phase of a moving target is subject to several psychophysical distortions. At the beginning of a constant motion, observers estimate velocity as being higher than in later phases of the motion; thus, they have the impression of a deceleration. Consequently, when a motion that accelerates in the initial phase is presented, observers judge it as having a constant velocity (e.g., Brouwer, Brenner, & Smeets, 2002; Runeson, 1974; Tayama, 2004). Another perceptual distortion is that target discrimination is impaired for targets presented at initial positions of motion onset (Ansorge, Carbone, Becker, & Turatto, 2010; Müsseler & Aschersleben, 1998).

The present study is concerned with two opposed localization errors when observers were asked to indicate the initial position of a moving target. Either they mislocalized the target’s onset position consistently in the direction of motion (the Fröhlich effect; Fröhlich, 1923; for a recent overview, see Kerzel, 2010), or the target’s onset was consistently mislocalized in the direction opposite to motion (the onset repulsion effect; cf. Thornton, 2002; see also Actis-Grosso & Stucchi, 2003; Hubbard & Motes, 2002; Hubbard & Ruppel, 2011; Kerzel, 2010).

**Fig. 1:**
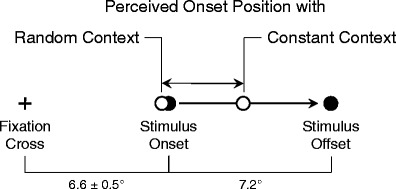
Stimulus presentation and stimulus perception in the experiments (cf. also Müsseler & Kerzel, 2004). A moving target appeared to the left or to the right of fixation. Perceived starting positions were in the direction of motion in constant-context conditions and (at least) essentially reduced in random-context conditions

In an effort to explain the discrepancy between the two sets of observations, Müsseler and Kerzel (2004; see also Müsseler, Stork, & Kerzel, 2008) found that the spatial predictability of the target onset position varied between studies. When the spatial onset predictability was high, targets appeared at a fairly constant eccentricity to the left and right of fixation (constant-context condition; cf. Müsseler & Aschersleben, 1998). When spatial onset predictability was low, the target’s onset was random within a large square field centered on fixation (random-context condition; cf. Thornton, 2002). In the experiments of Müsseler and Kerzel, the same onset positions as in the constant-context condition were presented on a fraction of the trials in the random-context condition (one sixth of the trials). Only these trials in the random-context condition were compared with the trials in the constant-context condition. The result was that onset mislocalizations were in the direction of motion in the constant-context condition (i.e., the Fröhlich effect) and, by trend, were opposite the true onset position in the random-context condition (i.e., the onset repulsion effect). The fact that Müsseler and Kerzel did not observe a statistically reliable onset repulsion effect might originate from the still above-chance level of left or right presentations in the random-context condition. In any case, what the experiments of Müsseler and Kerzel revealed was that localization of the adjusted onset position varied strongly with trial context (Fig. 1). Perceived starting positions were in the direction of motion in constant-context conditions and (at least) essentially reduced in random-context conditions. The mechanism underlying this difference between context conditions was explained mainly post hoc and was not addressed experimentally.

In the present study, four assumptions were tested addressing the different localizations in the constant-context and random-context conditions. First, we examined an overcompensation mechanism, which has been suggested by several authors to account for the difference between the Fröhlich and onset repulsion effects (cf. Actis-Grosso & Stucchi, 2003; Kerzel & Gegenfurtner, 2004; Müsseler & Kerzel, 2004; Thornton, 2002). The assumption is that when positional uncertainty is high, as is the case in the random-context condition, observers may notice a target relatively late and, with every new trial, become aware of a possible localization error. To avoid this error, they may overcompensate for their judgment and point to positions opposite to motion.

An experiment by Müsseler and Kerzel (2004, Experiment 4) cast first doubts on the overcompensation assumption. The authors examined how observers’ localization responses changed within an experiment. With regard to the overcompensation mechanism, a reduction or even reversion of the localization error should develop during the experiment in the random-context condition. Only when observers become aware of the possible localization error should they compensate for it. However, the findings showed an adaptation in the constant-context condition: At the beginning of the experiment, the localization error was small and did not differ between context conditions, but the localization error increased with the constant-context condition after only 22 trials, while the localization error in the random-context condition remained quite stable throughout the experiment. Obviously, there was some kind of adaptation only on constant-context trials.

Nevertheless, the overcompensation account should be put to another test. With regard to the overcompensation mechanism, the difference between context conditions is assumed not to be a perceptual one but to result from the tendency in the adjustment phase to correct for the possible spatial error. Consequently, an overcompensation mechanism should mainly affect a localization task, but not a discrimination task. This was examined in Experiment 1.

Second, the present study examined whether the essential mislocalization in the constant-context condition originated from motion-based predictions. The mechanism we assumed bore reference to that in eye movement studies, which have revealed that saccades tend to overshoot the onset position when the observer experiences only directed movements at that position (Müsseler, Brinkmeier, & Stork, 2004; see also Krauzlis & Adler, 2001). The interesting question to examine was whether such predictive remapping develops only in the saccadic system or also in perception (cf., e.g., Krauzlis & Nummela, 2011; Nijhawan, 2008). Although stimuli were moving in both conditions, the constant-context condition might be predisposed to evoke more motion-based predictions than the random-context condition, since the known onset positions allow, for instance, direction of attention to future stimulus positions (Krauzlis & Nummela, 2011). To examine the role of a target’s motion in the estimation of future positions, in Experiment 2, we compared a condition in which only moving stimuli were presented to the left or right of fixation with a condition in which trials with moving stimuli were interspersed within the majority of trials with static stimuli. Our prediction was that the first condition would evoke pronounced mislocalizations, while the second condition would not (or would evoke less pronounced mislocalizations, at least).

Third, the study addressed possible sequence effects between trials. In the constant-context condition, trial repetitions (i.e., trials with onset positions in the same field of vision—i.e., at either left or right locations) are much more frequent than in the random-context condition. It may be that position judgments are affected by repetitions between trials—for instance, by inhibiting the last onset area. An easy way to examine this explanation is to analyze *n* trials as repetitions or nonrepetitions of *n*−1 trials. The data of Experiments 2 and 3 served to conduct this analysis.

Fourth, the study examined whether attentional mechanisms produced the observed difference between constant-context and random-context conditions. When stimuli always appeared at predictable left or right positions, as was the case in the constant-context condition, observers probably directed their attention to both positions in advance (parallel allocation of visual attention to, at least, two positions; cf. Awh & Pashler, 2000; Cave, Bush, & Taylor, 2010; Franconeri, Alvarez, & Enns, 2007; but see also Jans, Peters, & De Weerd, 2010). The spatial uncertainty of onset positions in the random-context condition did not allow a comparable allocation of attention. This suggests that attentional mechanisms are responsible for the difference between the conditions.

However, several studies have shown that directed attention usually improves spatial localization judgments (e.g., Bocianski, Müsseler, & Erlhagen, 2008, 2010; Tsal & Bareket, 1999, 2005; Tsal, Meiran, & Lamy, 1995; Yeshurun & Carrasco, 1999). Thus, from an attentional point oft view, the present disadvantage of the constant-context condition is counterintuitive. Note, however, that so far, the influence of attention on localization performance has been studied mainly with stationary targets, but the stimuli were moving in the localization studies relevant here. What can make the difference is that—in order to follow the target—moving stimuli might require a spatial disengagement from the previously attended positions. Since attended positions are likely only in the constant-context condition, this could have impaired position judgments. Experiment 3 examined, with a cuing paradigm, whether and to what extent attentional mechanisms modify localization judgments in the constant-context and random-context conditions.

## Experiment 1

The overcompensation account claims that in the random-context condition, observers notice a stimulus relatively late and that they become aware of the possible localization error. They overcompensate for it by pointing to positions opposite to motion (cf. Actis-Grosso & Stucchi, 2003; Kerzel & Gegenfurtner, 2004; Müsseler & Kerzel, 2004; Thornton, 2002). If this is correct, the better performance of the random-context condition should pertain only to a localization task, while a discrimination task, for instance, should remain unaffected.

In the present experiment, moving stimuli either started out as squares and changed to circles at five different positions on the motion trajectory or appeared as circles and did not change. Observers’ task was to discriminate whether or not they perceived a square during the motion of the target stimulus.

The overcompensation account would expect equal or worse discrimination performance in the random-context condition than in the constant-context condition. This result would point out a response bias, which compensates for a possible localization error in the adjustment phase (Müsseler & Kerzel, 2004; Müsseler et al., 2008). The contrary finding (i.e., the random-context condition shows better discrimination performance) cannot be explained by an overcompensation mechanism and would indicate a perceptual origin of the difference between context conditions.

### Method

#### Apparatus and stimuli

The experiments were run on a Macintosh computer with a 22-in. color CRT monitor (Iiyama Vision Master Pro 513, 100-Hz refresh rate, 1,024 × 768 pixels). Participants sat in a dimly lit room with their head placed on a chinrest 500 mm in front of the monitor and the line of gaze straight ahead. Stimulus presentation was controlled by the MATLAB Software Package using the Psychophysics (Brainard, 1997; Pelli, 1997) and Eyelink Toolbox extensions (Cornelissen, Peters, & Palmer, 2002).

The stimuli were presented on a light gray background of about 20.1 cd/m^2^. A black disk or a square of 0.7° visual angle with a luminance of 1.1 cd/m^2^ was used as a moving stimulus. The stimuli moved at a velocity of 26.7 °/s for 270 ms, yielding a trajectory length of 7.2°.

Two context conditions were compared. In the constant-context condition, the stimuli appeared always at 6.6° ± 0.5° to the left or to the right of the fixation cross and moved away from the fovea. In the random-context condition, the same applied for one third of the trials. For the other two thirds, the onset positions varied randomly within a square of 30.8° × 30.8° centered on the fixation cross. As in the constant-context condition, motion was always horizontal and foveofugal.

#### Procedure

A central fixation cross was visible throughout the experiment. Each trial started with an auditory warning signal. After a delay of 500 ms, the target stimulus appeared to the left or to the right of the fixation cross and moved horizontally outward toward the edge of the screen. The instruction stressed holding fixation while the target was moving. Observers’ task was to indicate with a keypress whether or not they perceived a square during stimulus presentation. On two thirds of the trials, stimuli started out as squares and changed to disks after the presentation of the 1st (i.e., after 10 ms), 3rd (30 ms), 5th (50 ms), 7th (70 ms), or 14th (140 ms) frame. On one third of the trials, a disk appeared from the beginning.

The number of trials was kept constant across the constant-context and random-context conditions. All participants worked through 2,160 trials split into four experimental blocks of 540 trials performed on two consecutive days at approximately the same time of day. Each 540-trial block lasted about 50 min, and participants were asked to pause for at least 15 min between blocks. Overall, the experiment lasted about 4 h, including the eye calibration and recalibration procedures, training sessions, and additional short breaks after every 45 trials.

#### Design

The constant-context and random-context conditions were presented in consecutive blocks to each participant, with the order of blocks counterbalanced between observers. With presentation time of squares (0, 10, 30, 50, 70, and 140 ms) as a factor, the experiment had a 2 × 6 design with repeated measurement on both factors.

#### Control of eye fixation

In the present and subsequent experiments, the horizontal position of the left eye was monitored with a head-mounted eye-tracking device (Eyelink II, SR Research). If a saccade greater than 1.5° was detected during the presentation of the stimuli, observers received a written feedback about not having preserved fixation, and the data for the corresponding trial were excluded from analysis. In the present experiment, the mean exclusion rate was 8.4 % in the constant-context condition and 7.8 % in the random-context condition.

#### Participants

Fifteen individuals (13 of them female) between 19 and 26 years of age (*M* = 21.8 years) received course credit for participating in the experiment. All participants in the present and subsequent experiments reported normal or corrected-to-normal vision.

### Results and discussion

**Fig. 2:**
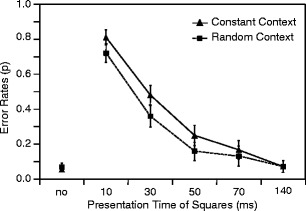
Mean error rates and between-participants standard errors for constant-context and random-context conditions as a function of presentation time of squares (Experiment 1)

Figure 2 shows the mean error rates in observers’ task to indicate whether or not they perceived a square during stimulus presentation in the constant-context and random-context conditions. In both conditions, error rates were computed only from identical trials—that is, from trials on which stimulus motion was horizontally presented at onset positions around ±6.6° eccentricity.

Results showed that the mean error rate was higher in the constant-context condition than in the random-context condition, especially when the squares were presented only for 10, 30, and 50 ms (mean error rates of .81, .48, and .25 vs. .72, .36, and .16). Accordingly, a 2 × 6 analysis of variance (ANOVA) showed a significant main effect of context condition, *F*(1, 14) = 9.92, *p* < .01, *η*_p_^2^ = .42, a significant main effect of presentation time of the squares, *F*(2.18, 30.53) = 87.46, *p* < .001, *η*_p_^2^ = .86, and a significant two-way interaction, *F*(2.26, 31.57) = 4.28, *p* < .05, *η*_p_^2^ = .23 (degrees-of-freedom corrections with regard to Greenhouse & Geisser, 1959). The differences between constant-context and random-context conditions were significant for the presentation times of the squares of 10 ms, *t*(14) = 2.12, *p* < .052, 30 ms, *t*(14) = 3.36, *p* < .01, and 50 ms, *t*(14) = 3.20, *p* < .01 (always two-tailed).

The findings clearly demonstrated that with short presentation times of the squares, discrimination performance was far better in the random-context condition than in the constant-context condition. Thus, the difference between both conditions was visible not only in a localization task, but also in a discrimination task. Obviously, observers can localize and discriminate stimuli better when presented in random-context conditions. As a consequence, an overcompensation mechanism, as has been assumed to explain the difference between conditions with a localization task, is not very likely.

## Experiment 2

The present experiment examined whether the mislocalization originated from motion-based predictions, which develop mainly in the constant-context condition. To examine this idea, in the constant-context condition, localization judgments were again gathered with moving stimuli and predictable starting positions presented at 6.6° to the left or to the right of fixation. Since this condition was ideal for predictions of the motion trajectory, we expected considerable localization errors in motion direction. In contrast, in the random-context condition, trials with moving stimuli were interspersed within the majority of trials with static stimuli, which also appeared at 6.6° eccentricity. Note that spatial predictability was high in both conditions; here, the term *random context* now refers to the mixed presentation of static and dynamic stimuli. In this condition, observers should not form—or at least form fewer – expectations about stimulus motions. Consequently, the predictive account would lead one to expect reduced localization errors for moving stimuli in this condition.

### Method

#### Stimuli, design, and procedure

The stimuli, design, and procedure were the same as in Experiment 1, except for the following changes. Stimulus presentation occurred only in the spatial configuration of the constant-context condition of Experiment 1; that is, stimulus onset positions were always at 6.6° ± 0.5° to the left or right of fixation. In the constant-context condition, only moving disks were shown, while in the random-context condition, moving disks appeared on only one third of all trials. For the remaining two thirds of the trials, static disks were presented for one frame (10 ms). Thus, in the random-context condition, moving stimuli were only interspersed within the presentations of static stimuli.

An auditory signal was presented 1 s after stimulus presentation, and a cursor appeared at the central position of the screen. The observers’ task was to adjust the cursor with the mouse to the position where they had first perceived the moving or the static disk. The adjustment cursor was a replica of the target disk and was visible only during the adjustment phase. While moving the cursor to the perceived starting position, observers were allowed to move their eyes freely. Adjustment was finally confirmed with a mouse click, which initiated the next trial after a 1-s delay.

All participants worked through 120 trials split into experimental blocks of 12 trials. Overall, the experiment lasted about half an hour, including the eye calibration procedure, training session, and additional short breaks between blocks.

In the present experiment, the mean exclusion rate of trials with eye movement errors was 7.4 % in the constant-context condition and 12.9 % in the random-context condition.

#### Participants

Twelve fresh individuals (10 of them female), between 20 and 24 years of age (*M* = 22.4 years), were paid to participate in Experiment 2.

### Results and discussion

Mean differences between the adjusted and the true onset positions were calculated for each observer and condition. Positive values indicate mislocalizations in the direction of motion, whereas negative values indicate mislocalizations opposite to the motion direction. For static stimuli, negative values indicate that target positions were perceived as being closer to the fixation point than they actually were.

**Fig. 3:**
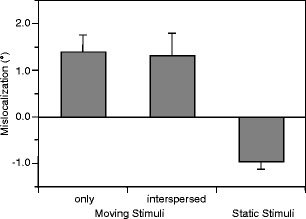
Mean mislocalizations and between-participants standard errors for the first position of moving stimuli (left bars) and for the position of static stimuli (right bar). Positive values indicate errors in the direction of motion. For static stimuli, negative values indicate perceived target positions closer to the fixation point than the target actually was (Experiment 2)

Figure 3 shows essential mislocalization in the direction of motion (i.e., the Fröhlich effect) in the constant-context condition (only moving stimuli; *M* = 1.40°), *t*(11) = 3.90, *p* < .01, and random-context condition (interspersed moving stimuli; *M* = 1.32°), *t*(11) = 2.80, *p* < .05. However, contrary to the assumptions of the adaptation account, the difference between the two conditions was far from significance, *t*(11) = 0.31, n.s. This result suggested that predictions of future targets’ positions on trajectories are not likely to explain the pronounced mislocalization in the constant-context condition but, obviously, predictions of onset positions are.

As a further finding of the experiment, static stimuli were perceived as being considerably closer to the fixation point than they actually were (*M* = −0.95°), *t*(11) = 5.56, *p* < .001. This result replicated previous findings (e.g., Stork, Müsseler, & van der Heijden, 2010; van der Heijden, van der Geest, de Leeuw, Krikke, & Müsseler, 1999; Wright, Morris, & Krekelberg, 2011) and indicated that the amount of the Fröhlich effect is rather underestimated than overestimated.

The trials in the constant-context condition served also to examine whether the location of stimulus presentations on the preceding *n*−1 trials exerted an influence on the mislocalization. Either stimulus presentations between trials were made at the same location (left or right side of fixation), or the location changed (from left to right or right to left side of fixation). Mislocalizations were 1.37° when the presentation location between trials did not change and 1.45° when the presentation location changed. The difference was also far from significance, *t*(11) = 0.61, n.s. Thus, the location of stimulus presentations on the preceding *n*−1 trials did not affect the mislocalization, but the analysis was repeated in Experiment 3 to confirm the result.

## Experiment 3

It is plausible to assume that observers directed their attention to the two onset positions in the constant-context conditions. As was mentioned in the Introduction, however, the present disadvantage for localization performance in the constant-context condition is counterintuitive from an attentional point oft view. The assumption that must be added is that—contrary to stationary stimuli—moving stimuli might require a spatial disengagement from the attended positions, which could impair position judgments in the constant-context condition. If this is correct, presenting an exogenous cue should elicit a similar mechanism, which could also impair localization judgments in random-context conditions. This was examined in the present experiment. For one group of participants, the moving stimuli in both constant and random contexts were preceded by a local visual cue, while another group of participants saw moving stimuli without a cue in both conditions.

Müsseler and Aschersleben (1998, Experiment 4) already conducted an experiment with constant-context conditions in which they presented a cue above and below the location where the horizontally moving stimulus appeared. The result was that the cue decreased the Fröhlich effect by only 0.4°, which suggested that the cue improved the processing of the moving stimulus (see also Ansorge et al., 2010). However, the Fröhlich effect was far from vanishing alltogether. Thus, even when a cue improved the processing of the moving stimulus, the critical question in the present experiment was whether the localization error would increase with presentation of the cue in the random-context conditions.

### Method

#### Stimuli and procedure

The stimuli and procedure were the same as in Experiment 1, except for the following changes. In the constant-context and random-context conditions, only moving disks were presented. Moving stimuli were presented with or without a visual cue. Presentation duration of the cue was 150 ms, and cues consisted of thin vertical lines with a width of 0.13° and a length of 0.7°, which were presented 0.8° above and below the location where the horizontally moving target appeared. Moving stimulus presentation started 130 ms after cue offset; thus, the stimulus onset asynchrony (SOA) between the cue and moving stimulus was 280 ms.

In the present experiment, the mean exclusion rate of trials with eye movement errors was 5.4 % in the constant-context condition and 5.3 % in the random-context condition.

#### Participants and design

Sixteen students (15 of them female), between 19 and 23 years of age (*M* = 20.3 years) were paid to participate in the experiment. They were randomly assigned to two groups. For one group of participants, the moving stimuli in both the constant-context and random-context conditions were preceded by the presentation of the visual cue, while the other group of participants saw moving stimuli without a cue in both conditions. Thus, the experiment was based on a 2 (groups with vs. without cue presentation) × 2 (constant-context vs. random-context condition) mixed design.

### Results and discussion

**Fig. 4:**
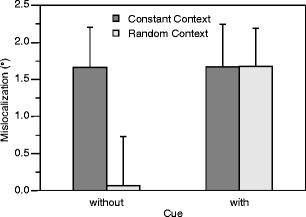
Mean mislocalizations and between-participants standard errors for the first position of moving stimuli. Positive values indicate errors in the direction of motion (Experiment 3)

The results are shown in Fig. 4. When no cue was presented, mean localization errors were 1.66° in the constant-context condition and 0.06° in the random-context condition. This result replicated successfully the difference between constant-context and random-context conditions in previous studies (Müsseler & Kerzel, 2004; Müsseler et al., 2008). However, when a cue was presented, mean localization errors were 1.66° in the constant-context condition and, as was expected, equally high at 1.67° in the random-context condition.

A 2 × 2 ANOVA confirmed the pattern of results statistically. The interaction was significant, *F*(1, 14) = 6.61, *p* < .05, *η*_p_^2^ = .32,. Additionally, a main effect of context condition was observed, *F*(1, 14) = 6.47, *p* < .05, *η*_p_^2^ = .32, whereas the main effect of cuing was not significant. Thus, the unexpected finding of the present experiment was somewhat contrary to what was observed in the study of Müsseler and Aschersleben (1998, Experiment 4; see also Whitney & Cavanagh, 2000); localization errors were not generally reduced in the with-cue conditions. It may be that the present between-participants design was not appropiate to evoke the observed small reduction of the Fröhlich effect. Another possibility is that processing was affected by the different SOAs between the cue and moving stimulus. The SOA was 125 ms in the study of Müsseler and Aschersleben, while it was 280 ms in the present experiment. For the 125-ms SOA, excitation at the cued location might dominate processing, whereas for the 280-ms SOA, inhibitory processes might come into play (cf. inhibition of return; Lupiàñez, 2010; Posner & Cohen, 1984).

Irrespective of that, the main result of the present experiment is that the localization error increased in the random-context conditions when a cue was presented. Moreover, the presentation of the cue in the random-context conditions elicited an error that is comparable in size to the localization error in the constant-context conditions. This is clear evidence that attentional factors contribute to the localization error observed in constant-context conditions.

Again, the trials in the constant-context condition served to examine whether the localization error varied with the location of stimulus presentations on the preceding *n*−1 trials. Localization errors were 1.58° when the presentation location between trials did not change and 1.69° when the presentation location changed. The difference was again far from significance, *t*(7) = 0.60, n.s. Thus, we can conclude that the essential localization error in the constant-context conditions did not result from the repetition of stimulus presentations at the same location.

## General discussion

The present study was concerned with the question of how trial context accounts for the perceived mislocalizations of the onset position of a moving stimulus. Three of four possible explanations proved to be not very likely. First, the overcompensation account claims that in the random-context condition, observers notice the target relatively late and, to avoid a localization error, overcompensate for it by pointing to positions opposite to motion. However, Experiment 1 demonstrated that the better localization performance in random-context conditions came along with a better discrimination performance, which is evidence against the notion that observers notice the target relatively late in random-context conditions. Second, since trial repetitions were much more frequent in the constant-context than in the random-context conditions, the increased localization errors in the constant-context conditions could have resulted from an inhibition mechanism for the area where the target had been presented on the preceding trial. To examine this suggestion, we analyzed *n* trials as repetitions or nonrepetitions of *n*−1 trials*.* However, the analysis of the data of Experiments 2 and 3 revealed no evidence for this suggestion. Third, Experiment 2 revealed that the constant-context condition showed mislocalizations comparable to those in a condition in which trials with moving stimuli were interspersed within the majority of trials with static stimulus presentations. This result suggested that motion-based predictions within the target’s trajectory were not likely to explain the essential mislocalizations in the constant-context condition but, obviously, the predictions of onset positions were.

What remained is the main finding of Experiment 3: When a cue preceded motion onset, the localization error in the random-context condition increased in size relative to the localization error in the constant-context condition. The function of a visual cue is usually seen as directing attention to a position in order to improve the processing of a subsequently presented target. Since, in the constant-context conditions, observers had also probably allocated their attention to the positions where the target would appear, the approximation in localization performances in the constant-context condition and random-context condition with cue presentation comes as no surprise. What is suprising is that localization performance was worse with allocated attention. Before this issue is discussed, what is to be said about Experiments 1 and 2 when the attentional position is taken up?

In Experiment 1, feature discrimination was worse in the constant-context conditions, although it was equally likely that observers would allocate their attention to the left and right positions at which the targets were presented. Worse discrimination performance (Experiment 1) went hand in hand with worse localization performance (Experiment 3). Thus, the results of Experiments 1 and 3 are consistent with each other. In Experiment 2, localization performance proved to be independent of whether only moving stimuli were presented or trials with moving stimuli were interspersed within the majority of trials with static stimuli. Since, in both conditions, stimuli were presented only to the left and right of fixation, spatial allocation of attention was likely comparable. As a consequence, the processing of only moving stimuli or interspersed moving stimuli should be affected by comparable attentional mechanisms, and this is what the results showed. The main difference between Experiments 2 and 3 seems to be that in Experiment 2, top-down attentional mechanisms took effect, while in Experiment 3, attentional allocation was aroused by bottom-up mechanisms.

At present, we can only speculate about what the attentional mechanism looks like that causes bad performance with moving stimuli. In the following, we will show that only one additional assumption in a dynamic field model, which already takes into account the positive effects of attention on localization performance with static stimuli, is needed (cf. Bocianski et al., 2008, 2010). The model, originally developed to investigate interaction effects observed in neural populations of the cat primary visual cortex (Jancke et al., 1999) and then successfully applied to perceptual mislocalizations with moving stimuli (for an overview, see Jancke & Erlhagen, 2010), assumes a network of excitatory and inhibitory activities that are tuned to positions in visual space. In accordance with the center-to-surround organization of receptive fields, activities integrate information over a large area. Therefore, in response to an afferent input, a spatial spread of activation is assumed that interacts with new incoming information and, thus, modifies suprathreshold activity. Similarly, it has been suggested that spreading subthreshold activation may constitute a neural correlate of a cue-induced attentional mechanism that alters the processing of spatial information (Kirschfeld & Kammer, 1999; Steinman, Steinman, & Lehmkuhle, 1995). Clearly, this suggestion assumes a stimulus-driven distortion of spatial information.

In a recent study, the dynamic field model was further extended by integrating a top-down directed mechanism (Bocianski et al., 2010). It was assumed that the blockwise presentation of a target at fixed positions (e.g., at 6° to the left and right of fixation, as in the present experiments) modulates the attentional baseline by arousing a peak at attended locations and by suppressing all other locations (for neural evidence of top-down modulation of the attentional baseline activity, see, e.g., Bestmann, Ruff, Blakemore, Driver, & Thilo, 2007; Smith, Singh, & Greenlee, 2000). If a static target is presented in the attended area, this leads to an improvement of localization precision (cf. Bocianski et al., 2010; Tsal & Bareket, 1999, 2005; Tsal et al., 1995; Yeshurun & Carrasco, 1999). However, when a fast moving target is presented in the attended area, the only assumption to add is that the target might have left the region of the attentional peak already before a suprathreshold activity was reached. Moreover, the new incoming information of the target may interact within the suppressed area, which could additionally impair localization performance. In a sense, the postulated mechanism is similar in accounts of the effects of spatial disengagement from previously attended positions. Certainly, this idea needs further experimentation.

Note that the ideas presented here cannot account for the onset repulsion effect, according to which the targets’ onset is consistently mislocalized opposite to motion (Thornton, 2002). In recent experiments, Hubbard and Ruppel (2011) examined, with implied motions, the effects of cuing on the onset repulsion effect. They found that mislocalization was diminished and or even vanished with the presentation of a cue. However, in this study, implied motion was induced by presenting stimuli at five positions, with interstimulus intervals of 250 ms each, resulting in an overall presentation time of 2,500 ms. Motion in the present study was much faster, with an overall presentation time of only 270 ms. This let us assume that the onset repulsion effect reflects a higher cognitive process in which the dynamics of the external environment are incorporated into the dynamics of cognitive representations.
